# The relationship between thyroid dysfunction, cardiovascular morbidity and mortality in type 2 diabetes: The Fremantle Diabetes Study Phase II

**DOI:** 10.1007/s00592-022-01969-x

**Published:** 2022-09-09

**Authors:** S. A. Paul Chubb, Kirsten E. Peters, David G. Bruce, Wendy A. Davis, Timothy M. E. Davis

**Affiliations:** 1 Medical School, Faculty of Health and Medical Sciences, Fremantle Hospital, University of Western Australia, PO Box 480, Fremantle, WA 6959 Australia; 2 PathWest Laboratory Medicine Western Australia, Fiona Stanley Hospital, Murdoch, WA Australia

**Keywords:** Thyroid disease, Type 2 diabetes, Cardiovascular disease, Mortality

## Abstract

**Aims:**

It is uncertain whether subclinical thyroid dysfunction is associated with cardiovascular disease (CVD) events and mortality in people with type 2 diabetes. The aim of this study was to determine whether undetected thyroid disease increases the risk of incident CVD and death in type 2 diabetes.

**Methods:**

One thousand two hundred fifty participants with type 2 diabetes (mean age 65.3 years, 56.5% males, median diabetes duration 8.0 years) without known thyroid disease and not taking medications known to affect thyroid function were categorised, based on baseline serum free thyroxine (FT4) and thyrotropin (TSH) concentrations, as euthyroid, overt hypothyroid (increased TSH, low FT4), subclinical hypothyroid (increased TSH, normal FT4), overt thyrotoxic (decreased TSH, raised FT4) or subclinical thyrotoxic (decreased TSH, normal FT4). Incident myocardial infarction, incident stroke, all-cause and cardiovascular mortality were ascertained during a mean 6.2–6.7 years of follow-up.

**Results:**

Most participants with newly-detected thyroid dysfunction had subclinical hypothyroidism (77.2%) while overt/subclinical thyrotoxicosis was infrequent. Compared to participants with TSH 0.34–2.9 mU/L, those with TSH > 5.1 mU/L were not at increased risk of incident myocardial infarction (adjusted hazard ratio (95% confidence limits) 1.77 (0.71, 2.87)), incident stroke (1.66 (0.58, 4.78)), all-cause mortality (0.78 (0.44, 1.37)) or cardiovascular mortality (1.16 (0.38, 3.58)). Independent baseline associates of subclinical hypothyroidism included estimated glomerular filtration rate and systolic blood pressure.

**Conclusions:**

Subclinical hypothyroidism was not independently associated with CVD events or mortality in community-dwelling people with type 2 diabetes despite its associations with CVD risk factors, questioning strategies to identify and/or treat mild thyroid dysfunction outside usual care.

**Supplementary Information:**

The online version contains supplementary material available at 10.1007/s00592-022-01969-x.

## Introduction

Thyroid disease and biochemical thyroid dysfunction are found frequently in diabetes. In the case of hypothyroidism, 6–20% of people with type 2 diabetes have overt hypothyroidism (an increased serum thyrotropin (TSH) and low serum free thyroxine (FT4) concentration) and a further 10% have subclinical hypothyroidism (an increased serum TSH and normal serum FT4) [1]. Thyroid overactivity is much less common, with approximately 4% of people with type 2 diabetes found to have overt thyrotoxicosis (a decreased TSH and raised serum FT4) and the same percentage subclinical thyrotoxicosis (a decreased serum TSH and normal serum FT4) [1].

Overt hypothyroidism affects multiple organ systems. In particular it may cause or contribute to dyslipidaemia [2], hypertension [3], renal dysfunction [4], and cardiac/endothelial dysfunction [5], all of which increase cardiovascular disease (CVD) risk. Although representing relatively mild thyroid dysfunction, subclinical hypothyroidism is also associated with adverse CVD risk factors [4–6]. While general population studies of the incidence of CVD in subclinical hypothyroidism have had variable results, an individual participant data meta-analysis found significantly increased risk of CVD and death in those with raised TSH concentrations [7]. In the case of thyrotoxicosis, there is also evidence of an association between overt thyrotoxicosis and CVD events and cardiovascular death [8] and the balance of evidence suggests that this is also the case for subclinical thyrotoxicosis [9].

The CVD risk factors in people with thyroid dysfunction are also frequently found in those with type 2 diabetes, suggesting that mild thyroid disease may be an additional, and potentially treatable, risk factor in this setting. In addition, minor degrees of thyroid dysfunction can act in concert with the insulin resistance that is characteristic of type 2 diabetes to worsen dyslipidaemia [10]. However, the existing studies of the impact of subclinical hypothyroidism on CVD risk in people with type 2 diabetes have been carried out in clinic-based or selected samples of patients [11–13] and only rarely in community-based cohorts [14], while there have been no population-based studies of the prognostic implications of overt or subclinical thyrotoxicosis complicating type 2 diabetes.

Given this background, the aim of the present study was to examine whether overt and subclinical thyroid dysfunction detected in a community-based sample of well characterised adults with type 2 diabetes were associated with CVD risk factors, key CVD outcomes and mortality. We hypothesised that thyroid dysfunction would influence CVD risk and directly and/or indirectly increase macrovascular complications and death.

## Methods

### Participants

The present study included a subset of 1,250 participants with type 2 diabetes and no known history of thyroid disease from Phase II of the Fremantle Diabetes Study (FDS2), a prospective representative community-based study of people living with diabetes in the primary catchment area of Fremantle Hospital. The FDS2 cohort comprises 1,732 participants with diabetes, including 1,483 (85.6%) with type 2 diabetes based on age at diagnosis, nature of first presentation, treatment history, body mass index and evidence of ketonaemia, and, where available, autoantibody positivity, serum insulin/C-peptide concentrations, and genotyping for those at high risk for monogenic forms [15]. Detailed information regarding recruitment and classification of diabetes has been published [15].

Participants with known prevalent thyroid disease (*n* = 158), based on a history of hypothyroidism, Hashimoto’s thyroiditis, Graves’ disease, nodular goitre, taking thyroxine, liothyronine and/or anti-carbimazole or propylthiouracil, or who had a history of thyroidectomy or ^131^I therapy by self-report or ascertained from linkage with the Hospital Morbidity Data Collection (HMDC) through the Western Australia Data Linkage System (WADLS) [16], were excluded from analyses. An additional 75 participants were excluded, comprising those taking amiodarone or lithium (*n* = 5) with the risk of iatrogenic thyroid disease, those with missing medication data (*n* = 9), or without suitable blood samples (*n* = 9), and 52 who also participated in the Fremantle Diabetes Study Phase I (FDS1) but who were living outside the catchment area. The FDS2 protocol was approved by the Southern Metropolitan Area Health Service Human Research Ethics Committee. All subjects gave informed consent before participation.

Clinical and biochemical data.

All participants underwent a detailed assessment at study entry which provided a comprehensive range of clinical, biochemical and questionnaire data. Each participant was invited to return for biennial face to face assessments interspersed with biennial postal questionnaires. Serum TSH, FT4 and antibodies to thyroperoxidase (anti-TPO) were measured in baseline samples at the nationally accredited PathWest laboratories at Fremantle Hospital, Western Australia, by automated electrochemiluminescence immunoassays using reagents from Roche Diagnostics Australia (Castle Hill, NSW, Australia) and a Cobas E601 analyser. Between-run imprecision, expressed as coefficients of variation, were ≤ 4.7% for FT4, ≤ 3.5% for TSH and ≤ 8.1% for anti-TPO antibodies. Reference ranges were 0.34–5.1 mIU/L for serum TSH, 12–22 pmol/L for serum FT4 (derived from a study of blood donors in the Fremantle area) and < 35 U/L for anti-TPO (as recommended by the manufacturer). Standard care testing of serum glucose, HbA_1c_, serum urea and electrolytes, serum lipids, serum uric acid, liver function, urine albumin and creatinine, and full blood count were performed using standard automated methods at PathWest Fremantle Hospital. Chronic complications were ascertained using standard criteria [17].

The FDS2 cohort was followed from baseline (2008–2011) until end-December 2016 for mortality outcomes and to end-June 2016 for incident myocardial infarction (MI) and stroke using linkage through the WADLS. The WADLS includes all public and private hospitalisations and death registrations within WA. Relevant International Classification of Disease (ICD)-9-CM and ICD-10-AM diagnosis codes were used to identify prevalent and incident MI and stroke in the HMDC (MI: ICD-9-CM codes 410 and ICD-10-AM codes I21, I22; stroke: ICD-9-CM codes 430–432, 433.01, 433.11, 433.21, 433.31, 433.81, 433.91, 434.01, 434.11, 434.91 and, ICD-10-AM codes I60-I64). Causes of death specified on the death certificate or in a coroner’s report were reviewed independently by two study physicians (DGB and TMED) and classified as cardiovascular (cardiac, cerebrovascular or sudden death) or otherwise.

Undiagnosed prevalent hypo- and hyperthyroidism at baseline were defined by an abnormal TSH. An elevated TSH (> 5.1 mIU/L) defined overt hypothyroidism in participants with a subnormal FT4 (< 12 pmol/L), and subclinical hypothyroid in those with normal FT4. Those with a low TSH (< 0.34 mIU/L) and elevated FT4 (> 22 pmol/L) were considered overtly hyperthyroid, while a low TSH and normal FT4 was categorised as subclinical hyperthyroidism. Since thyroid function tests are not part of usual-care biochemical screening in type 2 diabetes, they were performed retrospectively in the present study and were thus not used as the basis for clinical management decisions.

### Statistical analysis

The statistical analyses were performed using SPSS for Windows (version 25; SPSS Inc., Chicago, IL, USA). Data are presented as proportions, mean ± standard deviation (SD), geometric mean (SD range) or median [interquartile range (IQR)] as appropriate. For independent samples, two-way comparisons for proportions were by Fisher’s exact test, for normally distributed variables by Student’s *t*-test and for non-normally distributed variables by Mann–Whitney *U*-test. Serum TSH and FT4 were analysed using categories based on the assay reference ranges (TSH < 0.34, 0.34–2.9, 3.0–5.1 and > 5.1 mIU/L; FT4 < 12, 12–22 and > 22 pmol/L). A conservative Bonferroni approach was used to adjust for multiple comparisons. All clinically plausible variables with *P* ≤ 0.20 in bivariable analyses were considered for entry into multivariable models. Multiple logistic regression (backward conditional, variable entry and exit set at *P* < 0.05 and *P* ≥ 0.05, respectively) was used to identify independent associates of prevalent hypothyroid disease. Determinants of all-cause and cardiovascular mortality, incident MI, and incident stroke were determined using Cox proportional hazards regression modelling (backward conditional, variable entry and exit set at *P* < 0.05 or *P* < 0.01 and *P* ≥ 0.05 or *P* ≥ 0.01, respectively, depending on the number of outcomes). In the survival analyses, the lowest two TSH categories were combined due to small cell numbers.

## Results

### Associates of baseline thyroid dysfunction

At baseline, the 1,250 participants with type 2 diabetes had a mean ± SD age of 65.3 ± 11.5 (range 16.4–97.1) years, 56.5% were males, and their median [IQR] diabetes duration was 8.0 [2.5–15.6] years. The baseline characteristics of participants by biochemically defined thyroid disease are shown in Table 1. Sixty-one participants had subclinical hypothyroidism had four had a TSH > 10.0 mU/L, the highest being 12.0 mU/L. In bivariable analyses, compared to those with normal serum TSH, those with subclinical hypothyroidism were older at baseline, more likely to be anti-TPO positive, had lower eGFR, and higher systolic blood pressure. Independent associates of subclinical hypothyroidism included anti-TPO positivity, systolic blood pressure, and eGFR (negatively) (Table 2). In bivariable analyses, the 15 participants with overt hypothyroidism had significantly higher prevalence of anaemia and anti-TPO positivity than the euthyroid participants. There were too few participants with overt hypothyroidism for multivariable analysis. There were no significant differences between the hyperthyroid and euthyroid participants, but the numbers of hyperthyroid participants were too low for any meaningful comparisons.

**Table 1:** Baseline characteristics of 1,250 type 2 diabetes participants by biochemically defined thyroid disease

	Euthyroidism	Subclinical hypothyroidism	Overt hypothyroidism	Subclinical/overt hyperthyroidism	*P-*value
Number (%)	1,171 (93.7)	61 (4.9)	15 (1.2)	3 (0.2)	
Age (years)	65.1 ± 11.5	69.3 ± 10.0*	64.5 ± 11.8	60.5 ± 7.5	0.042
Male (%)	56.5	57.4	53.3	33.3	0.88
Ethnic background (%)					0.42
Anglo-Celt	51.4	49.2	60.0	100.0	
Southern European	13.5	13.1	0.0	0.0	
Other European	7.3	8.2	6.7	0.0	
Asian	4.8	3.3	0.0	0.0	
Aboriginal	7.0	6.6	26.7	0.0	
Mixed/other	16.0	19.7	6.7	0.0	
Not fluent in English (%)	11.4	13.1	6.7	0.0	0.87
Education beyond primary level (%)	86.8	83.1	100.0	100.0	0.42
Currently married/de facto relationship (%)	63.6	59.0	60.0	100.0	0.61
Daily alcohol consumption (standard drinks)	0.1 [0.0–1.5]	0.1 [0.0–0.8]	0.1 [0.0–0.6]	0.0 [0.0–0.0]	0.081
Smoking status (%):					0.23
Never	43.2	50.0	46.7	66.7	
Ex-	45.4	45.0	26.7	33.3	
Current	11.4	5.0	26.7	0.0	
Age at diabetes diagnosis (years)	55.2 ± 12.1	58.4 ± 10.3	53.8 ± 16.1	52.8 ± 12.4	0.22
Diabetes duration (years)	8.0 [2.3–15.4]	10.0 [4.0–17.8]	8.0 [3.0–17.2]	1.0, 9.0, 13.0^	0.59
Diabetes treatment (%):					0.89
Diet/exercise alone	23.9	19.7	26.7	33.3	
Oral hypoglycaemic agents	54.4	54.1	46.7	66.7	
Insulin ± oral hypoglycaemic agents	21.6	26.2	26.7	0.0	
Fasting serum glucose (mmol/L)	7.2 [6.2–8.9]	6.7 [5.7–8.6]	7.3 [6.0–9.2]	7.5, 7.9, 7.9^	0.32
HbA_1c_ (%)	6.8 [6.2–7.7]	6.8 [6.3–7.6]	7.2 [6.2–7.6]	5.9, 6.7, 7.7^	0.98
HbA_1c_ (mmol/mol)	51 [44–61]	51 [45–60]	55 [44–60]	41, 50, 61	0.98
ABSI^¶^ (m^11/6^ kg^−2/3^)	0.081 ± 0.005	0.082 ± 0.005	0.083 ± 0.005	0.080 ± 0.008	0.72
Body mass index (kg/m^2^)	31.1 ± 5.9	31.9 ± 6.6	32.0 ± 5.7	35.7 ± 10.4	0.36
Centrally obese (defined by waist circumference (%))	70.3	72.1	73.3	100.0	0.89
Pulse rate (beats/min)	70 ± 12	71 ± 12	70 ± 12	69 ± 15	0.75
Systolic blood pressure (mmHg)	145 ± 21	155 ± 25**	160 ± 30	147 ± 19	0.001
Diastolic blood pressure (mmHg)	80 ± 12	82 ± 15	86 ± 13	81 ± 3	0.18
Blood pressure-lowering medication (%)	74.1	70.5	66.7	100.0	0.66
Atrial fibrillation (%)	3.9	9.8	0.0	0.0	0.16
Left ventricular hypertrophy (%)	2.1	1.6	0.0	0.0	> 0.99
Lipid-lowering medications (%)	69.2	72.1	60.0	100.0	0.65
Total serum cholesterol (mmol/L)	4.3 ± 1.1	4.3 ± 1.0	4.8 ± 1.2	3.9 ± 1.4	0.44
Serum HDL-cholesterol (mmol/L)	1.22 ± 0.33	1.25 ± 0.35	1.40 ± 0.51	1.43 ± 0.47	0.15
Serum triglycerides (mmol/L)	1.5 (0.9–2.6)	1.7 (1.1–2.6)	1.6 (0.8–2.9)	1.0 (0.8–1.2)	0.15
High sensitivity C-reactive protein (mg/L)	2.4 (0.8–7.3)	3.0 (0.9–10.6)	2.8 (1.4–5.5)	2.3 (0.9–6.4)	0.42
Serum vitamin B-12 (pmol/L)	333 (211–525)	336 (202–559)	294 (161–537)	461 (375–566)	0.45
Serum bicarbonate (mmol/L)	24 ± 2	24 ± 2	24 ± 4	26 ± 3	0.40
Plasma NT-proBNP (pmol/L)	75 (18–308)	114 (18–734)	97 (20–468)	82 (53–126)	0.16
Serum albumin (g/L)	44.1 ± 2.9	43.4 ± 3.8	41.1 ± 6.4**	43.3 ± 4.2	0.001
Serum gamma glutamyltransferase (U/L)	32 (15–65)	30 (16–57)	34 (12–91)	17 (14–21)	0.47
Aspirin use (%)	37.0	42.6	46.7	66.7	0.44
Proton pump inhibitor use (%)	20.5	29.5	33.3	33.3	0.14
Serum uric acid (mmol/L)	0.33 (0.25–0.43)	0.34 (0.27–0.41)	0.35 (0.29–0.44)	0.34 (0.33–0.35)	0.80
eGFR (CKD-EPI) (ml/min/1.73m^2^)	73.2 ± 22.8	61.0 ± 23.3***	67.0 ± 21.7	73.9 ± 30.3	0.001
eGFR (CKD-EPI) (%):					0.029
≥ 90 mL/min/1.73m^2^	26.3	11.5	6.7	33.3	
60–89 mL/min/1.73m^2^	44.3	41.0	53.3	33.3	
45–59 mL/min/1.73m^2^	17.5	24.6	13.3	33.3	
30–44 mL/min/1.73m^2^	7.6	14.8	26.7	0.0	
< 30 mL/min/1.73m^2^	4.4	8.2	0.0	0.0	
Urinary albumin:creatinine ratio (mg/mmol)	3.1 (0.8–11.6)	4.6 (0.7–29.2)	4.9 (0.7–34.6)	1.9 (0.8–4.5)	0.92
Peripheral sensory neuropathy (%)	57.9	63.9	53.3	33.3	0.63
Any diabetic retinopathy (%)	37.2	43.9	40.0	33.3	0.79
Peripheral arterial disease (%)	21.8	18.0	20.0	33.3	0.79
Cerebrovascular disease (%)	7.9	4.9	13.3	0.0	0.55
Stroke (%)	2.6	0	6.7	0.0	0.26
Coronary heart disease (%)	27.3	29.5	46.7	66.7	0.14
Myocardial infarction (%)	7.9	9.8	13.3	33.3	0.19
Heart failure (%)	5.6	13.1	0.0	0.0	0.12
Anemia (%)	10.2	13.1	40.0*	0.0	0.010
Charlson Comorbidity Index (%)					0.61
0	76.3	75.4	66.7	100.0	
1–2	16.6	16.4	13.3	0.0	
≥ 3	7.1	8.2	20.0	0.0	
Anti-TPO positivity (%)	5.0	19.7***	33.3*	0.0	< 0.001

Data are presented as percentages, mean ± SD (standard deviation), geometric mean (SD range), or median [IQR – interquartile range]. ^ The data for the three individuals in this group are shown. ^¶^A Body Shape Index is a more robust index of obesity than body mass index [32]. *P < 0.05, **P < 0.01, ***P < 0.001 vs euthyroid group, adjusted for multiple comparisons using Bonferroni correction. NT-proBNP, N-terminal pro B-type natriuretic peptide; eGFR, estimated glomerular filtration rate by CKD Epidemiology Collaboration equation; TPO, thyroid peroxidase antibody

**Table 2:** Multiple logistic regression model of independent associates of undiagnosed prevalent subclinical hypothyroidism in 1,232* participants with type 2 diabetes

Variable	OR (95% CI)	*P-*value
Anti-TPO-positivity	5.04 (2.50, 10.16)	< 0.001
Systolic blood pressure (increase of 10 mmHg)	1.15 (1.03, 1.29)	0.017
eGFR (increase of 1 mL/min/1.73m^2^)	0.98 (0.97, 0.99)	< 0.001

*Subclinical hypothyroidism (n = 61) vs euthyroidism (n = 1171); all clinically plausible variables with bivariable *P* ≤ 0.20 considered for entry into the most parsimonious model using backward conditional logistic regression (*P* < 0.050 for entry, *P* ≥ 0.050 for removal)

### Thyroid dysfunction and incident stroke and myocardial infarction

Among the 1,219 participants with no known thyroid disease and no stroke prior to enrolment, 42 had an incident stroke during 7,603 person-years (mean ± SD 6.2 ± 1.6 years) of follow-up. Among 1,149 participants with no known thyroid disease and no MI prior to enrolment, 103 had an incident MI during 7,129 person-years (mean ± SD 6.2 ± 1.7 years) of follow-up. There were no bivariable associations between incident stroke or MI and either TSH or FT4 categories (*P* ≥ 0.14; Supplementary Table 2). The most parsimonious Cox models of incident stroke and incident MI with TSH categories added are shown in Table 3. TSH categories did not add significantly to the models for either outcome (*P* ≥ 0.22). In particular, the incidence of stroke for those with a TSH < 3.0 mU/L was not statistically different to that for participants with a TSH 3.0–5.1 mU/L. No incident strokes or MIs occurred in those with a high FT4, and only three incident strokes and four incident MIs were recorded in those with low FT4, so FT4 categories were not added to the respective most parsimonious Cox models.

**Table 3:** TSH category added to most parsimonious Cox proportional hazards models of independent predictors of incident stroke and myocardial infarction, using time as the timeline, in participants with type 2 diabetes and no known history of thyroid disease, and of stroke or myocardial infarction, respectively

	HR (95% CI)	*P-*value
Stroke:		
Age (increase of 10 years)	2.02 (1.46, 2.80)	< 0.001
HbA_1c_ (increase of 1%/11 mmol/mol)	1.44 (1.20, 1.74)	< 0.001
Serum uric acid (increase of 0.1 mmol/L)	1.77 (1.29, 2.42)	< 0.001
TSH category* (mIU/L):		0.62
< 3.0 (reference)	1.00	
3.0–5.1	1.17 (0.58, 2.36)	0.67
> 5.1	1.66 (0.58, 4.78)	0.35
Myocardial infarction:		
Aboriginal	2.20 (1.20, 4.02)	0.011
Current smoking habit	2.38 (1.43, 3.96)	< 0.001
Diabetes duration (increase of 5 years)	1.22 (1.09, 1.36)	< 0.001
Ln(plasma NT-proBNP (pmol/L))^†^	1.32 (1.14, 1.51)	< 0.001
Peripheral arterial disease	1.66 (1.09, 2.54)	0.019
Coronary heart disease	1.53 (0.996, 2.36)	0.052
Heart failure	1.96 (1.04, 3.72)	0.038
TSH category* (mIU/L):		0.22
< 3.0 (reference)	1.00	
3.0–5.1	1.14 (0.74, 1.91)	0.58
> 5.1	1.77 (0.71, 2.87)	0.084

All clinically plausible variables with bivariable *P* ≤ 0.20 in Supplementary Table 2 were considered for entry into a parsimonious model (Cox proportional hazards model with backward conditional selection (entry *P* < 0.050 at entry and removal *P* ≥ 0.050)) with one-by-one removal of variables for consideration, the least significant first, until all variables remaining in the model had *P* < 0.050. TSH category was then entered. *The lowest TSH category (< 0.34 mIU/L) was combined with the low-normal category (0.34–2.9 mIU/L) due to low cell numbers. ^†^A 2.72-fold increase in x corresponds to an increase of 1 in the natural log (Ln) of x

### All-cause and cardiovascular mortality

Of 1,250 participants with type 2 diabetes and no known thyroid disease at baseline, 215 (17.2%) died, including 37 (17.2%) from cardiovascular causes during 9,520 person-years (mean ± SD: 6.7 ± 1.7 years) of follow-up. Baseline characteristics by vital status are summarised in Supplementary Table 1. There was no significant relationship between baseline serum TSH categories and subsequent all-cause and cardiovascular mortality (*P* = 0.12 and *P* = 0.18, respectively). Kaplan–Meier survival analysis (data not shown) showed borderline statistical significance for the effect of serum TSH categories on all-cause and cardiovascular mortality (log rank test, *P* = 0.057 and 0.044, respectively), but TSH categories did not add to the most parsimonious Cox model in each case (Table 4).

**Table 4:** Most parsimonious Cox proportional hazards models of independent predictors of all-cause and cardiovascular mortality, using time as the timeline, in 1,250 participants* with type 2 diabetes and no known history of thyroid disease, with TSH categories and FT4 categories added separately

	HR (95% CI)	*P-*value	HR (95% CI)	*P-*value
All-cause mortality:				
Age (increase of 10 years)	1.86 (1.58, 2.19)	< 0.001	1.95 (1.66, 2.30)	< 0.001
Male sex	2.03 (1.49, 2.76)	< 0.001	2.04 (1.50, 2.77)	< 0.001
Married/de facto	0.71 (0.53, 0.95)	0.023	0.71 (0.53, 0.96)	0.024
Current smoking habit	2.44 (1.65, 3.61)	< 0.001	2.46 (1.66, 3.66)	< 0.001
Systolic blood pressure (10 mmHg increase)	0.93 (0.88, 0.98)	0.010	0.92 (0.87, 0.98)	0.006
Pulse rate (10 beats/min increase)	1.31 (1.18, 1.45)	< 0.001	1.31 (1.18, 1.45)	< 0.001
Ln(hsCRP (mg/L))^†^	1.18 (1.04, 1.35)	0.012	1.19 (1.04, 1.36)	0.009
Ln(NT-proBNP (pmol/L))^†^	1.38 (1.23, 1.55)	< 0.001	1.38 (1.23, 1.55)	< 0.001
Serum albumin (increase of 1 g/L)	0.43 (0.27, 0.67)	< 0.001	0.48 (0.31, 0.74)	0.001
Anemia	1.64 (1.17, 2.32)	0.005	1.65 (1.18, 2.33)	0.004
eGFR < 30 ml/min/1.73m^2^	1.98 (1.18, 3.33)	0.010	1.95 (1.16, 3.25)	0.011
Peripheral arterial disease	1.37 (1.01, 1.85)	0.044	1.33 (0.98, 1.80)	0.072
TSH category** (mIU/L):		0.64		
< 3.0 (reference)	1.00			
3.0–5.1	0.92 (0.67, 1.28)	0.63		
> 5.1	0.78 (0.44, 1.37)	0.38		
Serum free T4 (pmol/L):				0.001
< 12			1.76 (1.003, 3.09)	0.049
12–22 (reference)			1.00	
> 22			6.54 (2.03, 21.07)	0.002
Cardiovascular mortality:				
ABSI^¶^ (0.001 m^11/6^ kg^−2/3^ increase)	1.10 (1.03, 1.18)	0.005		
Pulse rate (10 beats/min increase)	1.42 (1.13, 1.80)	0.003		
Ln(NT-proBNP (pmol/L))^†^	1.90 (1.50, 2.41)	< 0.001		
Peripheral arterial disease	2.57 (1.32, 5.01)	0.006		
TSH category** (mIU/L):		0.45		
< 3.0 (reference)	1.00			
3.0–5.1	1.59 (0.77, 3.27)	0.21		
> 5.1	1.16 (0.38, 3.58)	0.79		

*Five individuals who died from unknown causes were omitted from the analysis of cardiovascular mortality. All clinically plausible variables with bivariable *P* ≤ 0.20 in Supplementary Table 1 were considered for entry into a parsimonious model (Cox proportional hazards model with backward conditional selection; entry *P* < 0.050, removal *P* ≥ 0.050 for all-cause mortality and entry *P* < 0.010, removal *P* ≥ 0.010 for cardiovascular mortality) with one-by-one removal of variables for consideration, the least significant first, until all variables remaining in the model had *P* < 0.050 for all-cause mortality and *P* < 0.010 for cardiovascular mortality. TSH or FT4 category was then entered. **The lowest TSH category (< 0.34 mIU/L) was combined with the low-normal category (0.34–2.9 mIU/L) due to low cell numbers. ^¶^A Body Shape Index is a more robust index of obesity than body mass index [32]. ^†^A 2.72-fold increase in x corresponds to an increase of 1 in the natural log (Ln) of x

FT4 categories were bivariably associated with all-cause mortality (*P* = 0.024; Supplementary Table 1). After adjustment for the most parsimonious Cox model of all-cause mortality (Table 4), FT4 categories added significantly (trend *P* = 0.001). Compared with FT4 of 12–22 pmol/L (reference category; 197 deaths in 1188 participants (16.6%)), a low FT4 was associated with a 76% increased risk of death (15 deaths in 55 participants (27.3%)) and high FT4 with a greater than sixfold increased risk. However, there were only seven participants with high FT4, three (42.9%) of whom died, and so the data need to be interpreted with caution. As there were no cardiovascular deaths in those with a high FT4 and only one in those with low FT4, FT4 categories were not added to the most parsimonious Cox model for cardiovascular mortality.

## Discussion

In this study of well characterised, community-based people with type 2 diabetes and no known thyroid disease, we found that previously undiagnosed subclinical hypothyroidism was significantly and independently associated with a higher systolic blood pressure and impaired renal function. Despite these relationships, there were no independent statistically significant associations between serum TSH concentrations and incident stroke, MI, or cardiovascular or all-cause mortality over a more than six-year average follow-up period. Undetected thyrotoxicosis, whether overt or subclinical, was rare in our cohort. These findings have potential implications for type 2 diabetes management in that they do not support attempts to identify and/or treat mild thyroid dysfunction outside usual care.

The association of diastolic hypertension with overt hypothyroidism in the general population is well recognised [3]. Reports of a similar association in subclinical hypothyroidism have been inconsistent, with evidence of a modest association driven by studies in which the subclinically hypothyroid participants were significantly older than those who were euthyroid [18]. In the present study, participants with subclinical hypothyroidism were also older than those who were euthyroid, but the association of systolic blood pressure with subclinical hypothyroidism was robust after adjustment for age, suggesting that age is not the driver of this association in people with type 2 diabetes. Impaired renal function was also independently associated with subclinical hypothyroidism in the present study, as has been seen in other studies in diabetes [12, 19, 20]. In people with chronic kidney disease, there is evidence that those with persistent subclinical hypothyroidism have a greater rate of eGFR decline and a greater incidence of end-stage renal disease compared to those with transient subclinical hypothyroidism or who remain euthyroid [21]. If chronic subclinical hypothyroidism has a similar adverse impact on renal function in type 2 diabetes, which is itself a strong risk factor for chronic kidney disease, its detection and possible treatment may be more important than in the general population.

We found no evidence that an increased TSH was independently associated with subsequently increased cardiovascular or all-cause mortality. Both low and high FT4 were significantly associated with all-cause mortality in multivariable analyses but the numbers in these groups were very low (only seven participants had high FT4) which questions the clinical significance of this observation. Subclinical hypothyroidism was associated with neither all-cause nor cardiovascular mortality after adjustment for other variables in female participants with type 2 diabetes enrolled in the earlier FDS Phase I (FDS1) [14], in a clinic-based sample of people with type 2 diabetes [19], and in a small-scale study of patients with type 2 diabetes undergoing renal dialysis [11]. Taken together, these findings questions whether subclinical hypothyroidism has prognostic implications in type 2 diabetes.

There have been a range of studies of the association between subclinical hypothyroidism and mortality in the general population, the results of which have been inconsistent. One individual participant data meta-analysis found that subclinical hypothyroidism, although not associated with all-cause mortality, was associated with incident coronary heart disease (CHD) and with CHD mortality at TSH concentrations > 10 mU/L, especially if those starting thyroid replacement were omitted from the analysis [7]. More recent study-level meta-analyses found significant associations between overt or subclinical hypothyroidism and both all-cause mortality and incident CVD [22, 23]. These reports revealed a modest increased mortality risk associated with persistent subclinical hypothyroidism in the general population. Our observation that this is not the case in type 2 diabetes may reflect that people with diabetes managed in a well-resourced health system such as in Australia may be both screened and treated relatively intensively for comorbidities such as thyroid disease and more rigorously managed for CVD risk factors than people without diabetes.

We similarly found that an increased TSH was not a determinant of incident stroke or MI. A systematic review of general population studies found no association between stroke and either subclinical hypothyroidism or subclinical hyperthyroidism [24]. A subsequent individual participant data meta-analysis used the same studies as in the systematic review but included unpublished data from other studies which substantially increased the number of participants and thus study power. There was still no association overall, but younger participants with subclinical hypothyroidism were at significantly increased risk of stroke [25]. More recent, large population-based studies from Scandinavia have also found no independent association between subclinical hypothyroidism and stroke [26, 27]. Our observations are therefore in accord with larger studies involving predominantly participants without diabetes. Interestingly, reports of reduced stroke risk at higher TSH concentrations within the reference interval have been published [13], but we found no indication of a differential incidence of stroke in those with TSH < 3.0 mU/L or 3.0–5.1 mU/L.

Our observation that an increased TSH was not a determinant of incident MI is consistent with our previous observation from FDS1 of CHD in women with type 2 diabetes [28], as well as other studies of people with type 2 diabetes [11, 19] and the results from participants with diabetes and a TSH within the conventional reference interval in the Second Manifestations of ARTerial disease study [13]. General population studies have produced inconsistent results, but there may be an association between subclinical hypothyroidism and CHD at TSH levels > 10 mU/L [7, 27, 29, 30]. We interpret our results and those of other authors as indicating that the excess risk of CHD due to subclinical hypothyroidism in type 2 diabetes is minimal, if present at all.

The present study had limitations. The low numbers with subclinical hypothyroidism in our cohort may have limited our ability to draw conclusions regarding its relationship with endpoints, especially for the less common outcomes. We also had limited power to examine independent associates of overt hypothyroidism and the associated risk of death. Thyroid function was performed only at baseline and so we were unable to assess the mortality risk for those with transient versus persistent subclinical hypothyroidism. We did not measure serum free T3, a variable which may have clarified the status of the few participants with a high FT4. We were unable to adjust our analyses for incident thyroid replacement therapy or other interventions with implications for thyroid function as this information was not available to us for all participants. Such additional data may have strengthened associations with mortality, as shown in one meta-analysis [7]. There were too few participants with positive TPO antibodies among those with raised TSH to allow a meaningful analysis of the effect of this variable on mortality and cardiovascular outcomes, and too few Aboriginal people to assess the possible consequences of concomitant thyroid disease and type 2 diabetes in this vulnerable sub-group. The uptake of the glucagon-like peptide 1 receptor agonists and sodium-glucose co-transporter-2 inhibitors, classes of blood glucose-lowering therapies with glucose-independent benefits for cardiovascular disease, was relatively low in the FDS2 cohort (< 5% in each case at the end of the follow-up period) given that they were first introduced into Australia when the study was in progress. The strengths of the study are its representative, community-based sample and the comprehensive prospective data collection.

Although thyroid dysfunction affects a third or more of people with type 2 diabetes [1], routine biochemical screening for thyroid disease is not mentioned in many major national and international guidelines, and a recent thyroid-specific position paper recommends against screening in patients with type 2 diabetes [31]. This may be appropriate given that most of our patients in whom thyroid disease was newly detected (77.2%) had subclinical hypothyroidism, a condition without a clear relationship to CVD events and all-cause mortality in type 2 diabetes and for which there are no trial data indicating benefit of replacement therapy in preventing these outcomes [1]. Nevertheless, even in a relatively resource rich healthcare environment such as Australia, the present data suggest that in people with type 2 diabetes and coincident hypertension and/or renal impairment, a possible contribution of thyroid disease should be considered as part of clinical assessment.

## Supplementary Information

Below is the link to the electronic supplementary material.

Supplementary file1 (DOCX 48 KB)

## References

[1] Biondi B, Kahaly GJ, Robertson RP (2019). Thyroid dysfunction and diabetes mellitus: two closely associated disorders. Endocr Rev.

[2] Cappola AR, Ladenson PW (2003). Hypothyroidism and atherosclerosis. J Clin Endocrinol Metab.

[3] Danzi S, Klein I (2003). Thyroid hormone and blood pressure regulation. Curr Hypertens Rep.

[4] Meuwese CL, van Diepen M, Cappola AR (2019). Low thyroid function is not associated with an accelerated deterioration in renal function. Nephrol Dial Transplant.

[5] Biondi B, Klein I (2004). Hypothyroidism as a risk factor for cardiovascular disease. Endocrine.

[6] Asvold BO, Bjoro T, Nilsen TI, Vatten LJ (2007). Association between blood pressure and serum thyroid-stimulating hormone concentration within the reference range: a population-based study. J Clin Endocrinol Metab.

[7] Rodondi N, den Elzen WP, Bauer DC, Cappola AR, Razvi S, Walsh JP (2010). Subclinical hypothyroidism and the risk of coronary heart disease and mortality. JAMA.

[8] Boelaert K, Franklyn JA (2005). Thyroid hormone in health and disease. J Endocrinol.

[9] Collet TH, Gussekloo J, Bauer DC, den Elzen WP, Cappola AR, Balmer P (2012). Subclinical hyperthyroidism and the risk of coronary heart disease and mortality. Arch Intern Med.

[10] Chubb SA, Davis WA, Davis TM (2005). Interactions among thyroid function, insulin sensitivity, and serum lipid concentrations: the Fremantle Diabetes Study. J Clin Endocrinol Metab.

[11] Drechsler C, Schneider A, Gutjahr-Lengsfeld L, Kroiss M, Carrero JJ, Krane V (2014). Thyroid function, cardiovascular events, and mortality in diabetic hemodialysis patients. Am J Kidney Dis.

[12] Jia F, Tian J, Deng F, Yang G, Long M, Cheng W (2015). Subclinical hypothyroidism and the associations with macrovascular complications and chronic kidney disease in patients with Type 2 diabetes. Diabet Med.

[13] de Vries TI, de Valk HW, van der Graaf Y, de Borst GJ, Cramer MJM, Jaap Kappelle L (2019). Normal-range thyroid-stimulating hormone levels and cardiovascular events and mortality in type 2 diabetes. Diabetes Res Clin Pract.

[14] Chubb SA, Davis WA, Davis TM (2006). Subclinical hypothyroidism and mortality in women with type 2 diabetes. Clin Endocrinol (Oxf).

[15] Davis WA, Peters KE, Makepeace A, Griffiths S, Bundell C, Grant SFA (2018). The prevalence of diabetes in Australia: insights from the fremantle diabetes study phase II. Intern Med J.

[16] Holman CD, Bass AJ, Rouse IL, Hobbs MS (1999). Population-based linkage of health records in Western Australia: development of a health services research linked database. Aust N Z J Public Health.

[17] Davis TM, Bruce DG, Davis WA (2013). Cohort profile: the fremantle diabetes study. Int J Epidemiol.

[18] Ye Y, Xie H, Zeng Y, Zhao X, Tian Z, Zhang S (2014). Association between subclinical hypothyroidism and blood pressure–a meta-analysis of observational studies. Endocr Pract.

[19] Chen HS, Wu TE, Jap TS, Lu RA, Wang ML, Chen RL (2007). Subclinical hypothyroidism is a risk factor for nephropathy and cardiovascular diseases in Type 2 diabetic patients. Diabet Med.

[20] Zhou JB, Li HB, Zhu XR, Song HL, Zhao YY, Yang JK (2017). Subclinical hypothyroidism and the risk of chronic kidney disease in T2D subjects: a case-control and dose-response analysis. Medicine (Baltimore).

[21] Kim EO, Lee IS, Choi YA, Lee SJ, Chang YK, Yoon HE (2014). Unresolved subclinical hypothyroidism is independently associated with progression of chronic kidney disease. Int J Med Sci.

[22] Ning Y, Cheng YJ, Liu LJ, Sara JD, Cao ZY, Zheng WP (2017). What is the association of hypothyroidism with risks of cardiovascular events and mortality? A meta-analysis of 55 cohort studies involving 1,898,314 participants. BMC Med.

[23] Moon S, Kim MJ, Yu JM, Yoo HJ, Park YJ (2018). Subclinical Hypothyroidism and the risk of cardiovascular disease and all-cause mortality: a meta-analysis of prospective cohort studies. Thyroid.

[24] Chaker L, Baumgartner C, Ikram MA, Dehghan A, Medici M, Visser WE (2014). Subclinical thyroid dysfunction and the risk of stroke: a systematic review and meta-analysis. Eur J Epidemiol.

[25] Chaker L, Baumgartner C, den Elzen WP, Ikram MA, Blum MR, Collet TH (2015). Subclinical hypothyroidism and the risk of stroke events and fatal stroke: an individual participant data analysis. J Clin Endocrinol Metab.

[26] Langen VL, Niiranen TJ, Puukka P, Lehtonen AO, Hernesniemi JA, Sundvall J (2018). Thyroid-stimulating hormone and risk of sudden cardiac death, total mortality and cardiovascular morbidity. Clin Endocrinol (Oxf).

[27] Selmer C, Olesen JB, Hansen ML, von Kappelgaard LM, Madsen JC, Hansen PR (2014). Subclinical and overt thyroid dysfunction and risk of all-cause mortality and cardiovascular events: a large population study. J Clin Endocrinol Metab.

[28] Chubb SA, Davis WA, Inman Z, Davis TM (2005). Prevalence and progression of subclinical hypothyroidism in women with type 2 diabetes: the Fremantle Diabetes Study. Clin Endocrinol (Oxf).

[29] Razvi S, Weaver JU, Vanderpump MP, Pearce SH (2010). The incidence of ischemic heart disease and mortality in people with subclinical hypothyroidism: reanalysis of the Whickham Survey cohort. J Clin Endocrinol Metab.

[30] Walsh JP, Bremner AP, Bulsara MK, O'Leary P, Leedman PJ, Feddema P (2005). Subclinical thyroid dysfunction as a risk factor for cardiovascular disease. Arch Intern Med.

[31] National Institute for Health and Care Excellence (2019) Thyroid disease: assessment and management (NICE guideline NG145)

[32] Krakauer NY, Krakauer JC (2012). A new body shape index predicts mortality hazard independently of body mass index. PLoS ONE.

