# Supplementary material for: The relationship between thyroid dysfunction, cardiovascular morbidity and mortality in type 2 diabetes: The Fremantle Diabetes Study Phase II
Source: Acta Diabetol. 2022 Sep 9;59(12):1615–24. doi: 10.1007/s00592-022-01969-x (PMC9581822; doi:10.1007/s00592-022-01969-x)
Supplement: Supplementary file 1 — Supplementary file1 (DOCX 48 KB) [file 592_2022_1969_MOESM1_ESM.docx]

**Supplementary Table 1.** Baseline characteristics of 1,250 participants with type 2 diabetes and without known thyroid disease by vital status at 31^st^ December 2016.

|  | All-cause mortality (n=1,250) | | | | Cardiovascular mortality (n=1,245*) | | | |
| --- | --- | --- | --- | --- | --- | --- | --- | --- |
|  | Alive | Deceased | *P-*value | Alive or deceased from other causes | | Deceased | *P-*value |  |
| Number (%) | 1035 (82.8) | 215 (17.2) |  | 1208 (96.6) | | 37 (3.0) |  |  |
| Age (years) | 63.8±10.9 | 72.7±11.2 | **<0.001** | 65.1±11.4 | | 71.4±11.6 | **0.001** |  |
| Males (%) | 55.1 | 63.3 | **0.029** | 56.1 | | 62.2 | 0.51 |  |
| Ethnic background (%): |  |  | 0.11 |  | |  | 0.60 |  |
| Anglo-Celt | 50.8 | 54.9 |  | 51.5 | | 54.1 |  |  |
| Southern European | 13.0 | 14.4 |  | 13.4 | | <13.5^†^ |  |  |
| Other European | 7.5 | 6.5 |  | 7.4 | | <13.5^†^ |  |  |
| Asian | 5.1 | 2.3 |  | 4.6 | | <13.5^†^ |  |  |
| Aboriginal | 6.7 | 9.8 |  | 7.0 | | <13.5^†^ |  |  |
| Mixed/other | 16.8 | 12.1 |  | 16.1 | | <13.5^†^ |  |  |
| Not fluent in English (%) | 10.9 | 13.5 | 0.29 | 11.5 | | <13.5^†^ | 0.79 |  |
| Education beyond primary level (%) | 88.1 | 80.2 | **0.003** | 86.6 | | 91.7 | 0.62 |  |
| Currently married/*de facto* relationship (%) | 66.0 | 51.2 | **<0.001** | 63.7 | | 51.4 | 0.17 |  |
| Age at diabetes diagnosis (years) | 54.5±11.6 | 59.3±13.4 | **<0.001** | 55.3±12.1 | | 57.1±13.4 | 0.37 |  |
| Diabetes duration (years) | 7.4 [2.0-15.0] | 13.2 [5.0-19.0] | **<0.001** | 8.0 [2.3-15.5] | | 14.2 [5.5-19.6] | **0.005** |  |
| Diabetes treatment (%): |  |  | **0.005** |  | |  | 0.42 |  |
| Insulin ± OGLMs/non-insulin injectables | 20.3 | 29.3 |  | 21.5 | | 27.0 |  |  |
| Fasting serum glucose (mmol/L) | 7.2 [6.2-9.0] | 6.9 [6.0-8.7] | **0.031** | 7.2 [6.2-8.9] | | 6.9 [5.8-9.2] | 0.60 |  |
| HbA_1c_ (%) | 6.8 [6.2-7.7] | 6.9 [6.2-7.7] | 0.56 | 6.8 [6.2-7.7] | | 7.1 [6.4-8.1] | 0.43 |  |
| HbA_1c_ (mmol/mol) | 51 [44-61] | 52 [44-61] | 0.56 | 51 [44-61] | | 54 [46-65] | 0.43 |  |
| Pulse rate (beats/min) | 69±12 | 73±14 | **<0.001** | 70±12 | | 74±15 | 0.074 |  |
| A Body Shape Index (m^11/6^ kg^-2/3^) | 0.081±0.005 | 0.083±0.005 | **<0.001** | 0.081±0.005 | | 0.084±0.008 | 0.062 |  |
| Body mass index (kg/m^2^) | 31.4±5.9 | 30.2±6.3 | **0.010** | 31.2±6.0 | | 29.0±4.5 | **0.028** |  |
| Central obesity (defined by waist circumference (%)) | 71.2 | 67.1 | 0.25 | 70.6 | | 62.2 | 0.28 |  |
| Systolic blood pressure (mmHg) | 145±21 | 150±24 | **0.007** | 145.8±21.5 | | 153.0±27.9 | 0.13 |  |
| Diastolic blood pressure (mmHg) | 81±12 | 79±13 | **0.044** | 80.6±12.2 | | 80.3±11.6 | 0.89 |  |
| Blood pressure-lowering medication (%) | 72.7 | 79.9 | **0.032** | 73.9 | | 75.7 | >0.99 |  |
| ACE-inhibitor | 38.0 | 45.8 | **0.038** | 39.6 | | 29.7 | 0.31 |  |
| ARB | 32.5 | 29.9 | 0.52 | 32.2 | | 32.4 | >0.99 |  |
| Beta-blocker | 17.7 | 28.0 | **0.001** | 19.5 | | 18.9 | >0.99 |  |
| Calcium-blocker | 24.2 | 26.6 | 0.49 | 24.7 | | 27.0 | 0.70 |  |
| Diuretic | 28.7 | 35.5 | **0.049** | 29.7 | | 40.5 | 0.20 |  |
| Other | 4.7 | 5.6 | 0.60 | 4.7 | | <13.5^†^ | 0.42 |  |
| Atrial fibrillation (%) | 2.7 | 10.9 | **<0.001** | 3.9 | | <13.5^†^ | 0.063 |  |
| Left ventricular hypertrophy (%) | 1.7 | 3.8 | 0.057 | 2.0 | | <13.5^†^ | 0.54 |  |
| Lipid-lowering medications (%) | 68.9 | 71.0 | 0.57 | 68.9 | | 78.4 | 0.28 |  |
| Total serum cholesterol (mmol/L) | 4.4±1.1 | 4.3±1.0 | 0.19 | 4.3±1.1 | | 4.3±1.1 | 0.76 |  |
| Serum HDL-cholesterol (mmol/L) | 1.22±0.33 | 1.24±0.40 | 0.68 | 1.23±0.34 | | 1.25±0.36 | 0.63 |  |
| Serum triglycerides (mmol/L) | 1.6 (0.9-2.6) | 1.5 (0.9-2.6) | 0.51 | 1.5 (0.9-2.6) | | 1.4 (0.9-2.2) | 0.30 |  |
| High sensitivity C-reactive protein (mg/L) | 2.3 (0.8-7.0) | 3.0 (1.0-9.3) | **0.001** | 2.4 (0.8-7.3) | | 3.3 (1.1-10.2) | 0.099 |  |
| Serum vitamin B-12 (pmol/L) | 334 (212-526) | 330 (203-535) | 0.73 | 333 (211-526) | | 348 (211-572) | 0.57 |  |
| Serum bicarbonate (mmol/L) | 25±2 | 24±3 | **<0.001** | 24±2 | | 24±2 | 0.77 |  |
| Plasma NT-proBNP (pmol/L) | 67.4 (16.7-271.6) | 139.9 (30.4-643.2) | **<0.001** | 74.6 (17.8-312.9) | | 158.9 (31.6-800.0) | **0.002** |  |
| Serum albumin (g/L) | 44.2±2.9 | 42.9±3.5 | **<0.001** | 44.0±3.0 | | 43.1±3.5 | 0.062 |  |
| Serum gamma glutamyltransferase (U/L) | 31 (16-62) | 34 (14-82) | 0.24 | 31 (15-64) | | 36 (13-95) | 0.44 |  |
| Aspirin use (%) | 36.3 | 42.7 | 0.087 | 37.3 | | 43.2 | 0.49 |  |
| Proton pump inhibitor use (%) | 19.1 | 30.7 | **<0.001** | 21.0 | | 27.0 | 0.41 |  |
| Serum uric acid (mmol/L) | 0.33 (0.26-0.42) | 0.34 (0.25-0.47) | 0.23 | 0.33 (0.25-0.43) | | 0.36 (0.28-0.45) | 0.10 |  |
| eGFR (CKD-EPI) (ml/min/1.73m^2^) | 75.6±20.9 | 58.0±26.5 | **<0.001** | 73.0±22.7 | | 59.0±25.9 | **<0.001** |  |
| eGFR (CKD-EPI) categories (%): |  |  | **<0.001** |  | |  | **0.004** |  |
| ≥90 ml/min/1.73m^2^ | 43.3 | 21.4 |  | 40.2 | | 18.9 |  |  |
| 60-89 ml/min/1.73m^2^ | 46.0 | 40.5 |  | 45.1 | | 48.6 |  |  |
| <60 ml/min/1.73m^2^ | 10.7 | 38.1 |  | 14.8 | | 32.4 |  |  |
| Urinary albumin:creatinine ratio (mg/mmol)^†^ | 2.9 (0.8-10.7) | 5.1 (1.1-23.3) | **<0.001** | 3.2 (0.8-12.2) | | 5.0 (1.1-22.8) | **0.040** |  |
| Peripheral sensory neuropathy (%) | 54.6 | 74.8 | **<0.001** | 57.9 | | 62.2 | 0.74 |  |
| Any diabetic retinopathy (%) | 36.7 | 41.6 | 0.20 | 37.4 | | 42.9 | 0.60 |  |
| Peripheral arterial disease (%) | 19.0 | 34.6 | **<0.001** | 21.1 | | 40.5 | **0.008** |  |
| Cerebrovascular disease (%) | 6.6 | 13.5 | **0.001** | 7.5 | | 16.2 | 0.059 |  |
| Coronary heart disease (%) | 24.5 | 43.3 | **<0.001** | 27.2 | | 45.9 | **0.016** |  |
| Heart failure (%) | 4.2 | 14.4 | **<0.001** | 5.6 | | 16.2 | **0.019** |  |
| Anemia (%) | 7.4 | 26.0 | **<0.001** | 10.5 | | 10.8 | >0.99 |  |
| Charlson Comorbidity Index (%): |  |  | **<0.001** |  | |  | 0.074 |  |
| 0 | 80.7 | 54.9 |  | 76.7 | | 62.2 |  |  |
| 1-2 | 14.6 | 25.6 |  | 16.3 | | 24.3 |  |  |
| ≥3 | 4.7 | 19.5 |  | 7.0 | | 13.5 |  |  |
| Daily alcohol consumption (standard drinks) | 0.1 [0.0-1.2] | 0.1 [0.0-1.5] | 0.27 | 0.1 [0.0-1.2] | | 0.1 [0.0-1.5] | 0.98 |  |
| Smoking status (%): |  |  | **0.012** |  | |  | 0.11 |  |
| Current | 10.2 | 16.5 |  | 10.9 | | 19.4 |  |  |
| Anti-TPO positivity (%) | 6.2 | 5.1 | 0.64 | 6.0 | | 5.4 | >0.99 |  |
| Serum TSH (mIU/L) (%): |  |  | 0.12 |  | |  | 0.18 |  |
| <0.34 | 0.2 | 0.5 |  | 0.2 | | 0.0 |  |  |
| 0.34-2.9 | 72.0 | 64.7 |  | 71.2 | | 56.8 |  |  |
| 3.0-5.1 | 22.0 | 27.4 |  | 22.6 | | 32.4 |  |  |
| >5.1 | 5.8 | 7.4 |  | 6.0 | | 10.8 |  |  |
| Serum free T4 (pmol/L) (%): |  |  | **0.024** |  | |  | >0.99 |  |
| <12 | 3.9 | 7.0 |  | 4.5 | | 2.7 |  |  |
| 12-22 | 95.7 | 91.6 |  | 95.0 | | 97.3 |  |  |
| >22 | 0.4 | 1.4 |  | 0.6 | | 0.0 |  |  |

* Five individuals who died from unknown causes were omitted from the analysis of cardiovascular mortality. Data are presented as percentages, mean±SD (standard deviation), geometric mean (SD range), or median [IQR – interquartile range]; NT-proBNP, N-terminal pro b-type natriuretic peptide; ACE-I, angiotensin-converting enzyme inhibitor; ARB, angiotensin receptor blocker; eGFR, estimated glomerular filtration rate by CKD Epidemiology Collaboration equation; TPO, thyroid peroxidase antibody; TSH, thyrotropin; T4, thyroxine. ^†^Actual percentages suppressed to preserve confidentiality

**Supplementary Table 2.** Baseline characteristics of 1,219 and 1,149 participants, respectively, with type 2 diabetes and without known thyroid disease or a history of stroke or MI, respectively, by incident stroke and incident myocardial infarction (MI) status at 30^th^ June 2016.

|  | Incident Stroke (n=1,219) | | | Incident Myocardial Infarction (n=1,149) | | |
| --- | --- | --- | --- | --- | --- | --- |
|  | No Stroke | Incident Stroke | *P-*value | No MI | MI | *P-*value |
| Number (%) | 1177 (96.6) | 42 (3.4) |  | 1046 (91.0) | 103 (9.0) |  |
| Age (years) | 64.9±11.4 | 72.1±9.2 | **<0.001** | 64.5±11.2 | 67.1±12.8 | 0.052 |
| Males (%) | 56.2 | 57.1 | >0.99 | 54.7 | 51.5 | 0.54 |
| Age at diabetes diagnosis (years) | 55.1±12.0 | 58.9±12.3 | 0.061 | 55.2±11.9 | 52.4±13.9 | **0.049** |
| Diabetes duration (years) | 8.0 [2.1-15.4] | 12.6 [6.0-17.7] | **0.007** | 7.4 [2.0-15.0] | 15.0 [6.0-20.0] | **<0.001** |
| Ethnic background (%): |  |  | 0.25 |  |  | **0.011** |
| Anglo-Celt | 51.5 | 50.0 |  | 51.3 | 47.6 |  |
| Southern European | 13.2 | 14.3 |  | 13.4 | 15.5 |  |
| Other European | 7.6 | <11.9^†^ |  | 7.9 | 4.9 |  |
| Asian | 4.6 | <11.9^†^ |  | 5.1 | <4.9^†^ |  |
| Aboriginal | 7.4 | <11.9^†^ |  | 6.0 | 16.5 |  |
| Mixed/other | 15.8 | 23.8 |  | 16.3 | 11.7 |  |
| Not fluent in English (%) | 11.3 | 11.9 | 0.81 | 11.8 | 12.6 | 0.75 |
| Education beyond primary level (%) | 87.1 | 90.5 | 0.64 | 87.2 | 83.7 | 0.35 |
| Currently married/*de facto* relationship (%) | 64.0 | 52.4 | 0.14 | 64.4 | 51.5 | **0.010** |
| A Body Shape Index (m^11/6^ kg^-2/3^) | 0.081±0.005 | 0.083±0.006 | **0.024** | 0.081±0.005 | 0.083±0.006 | **0.002** |
| Body mass index (kg/m^2^) | 31.2±6.1 | 30.2±4.3 | 0.16 | 31.3±6.0 | 30.7±6.1 | 0.29 |
| Central obesity (defined by waist circumference (%)) | 70.4 | 73.8 | 0.73 | 70.7 | 74.8 | 0.43 |
| Diabetes treatment (%): |  |  | 0.33 |  |  | **0.001** |
| Diet/exercise alone | 24.0 | 14.3 |  | 24.9 | 11.7 |  |
| Oral hypoglycemic agents | 54.5 | 59.5 |  | 55.0 | 56.3 |  |
| Insulin±Oral hypoglycemic agents | 21.5 | 26.2 |  | 20.1 | 32.0 |  |
| Fasting serum glucose (mmol/L) | 7.2 [6.2-8.9] | 7.2 [6.3-10.5] | 0.35 | 7.2 [6.2-8.9] | 6.9 [5.9-9.9] | 0.83 |
| HbA_1c_ (%) | 6.8 [6.2-7.7] | 7.4 [6.4-8.6] | **0.031** | 6.8 [6.2-7.6] | 6.9 [6.2-8.5] | 0.22 |
| HbA_1c_ (mmol/mol) | 51 [44-61] | 57 [46-70] | **0.031** | 51 [44-60] | 52 [44-69] | 0.22 |
| Blood pressure-lowering medication (%): | 73.0 | 92.9 | **0.002** | 72.2 | 71.8 | >0.99 |
| ACE-inhibitor | 38.5 | 50.0 | 0.15 | 37.5 | 35.0 | 0.67 |
| ARB | 32.0 | 33.3 | 0.87 | 31.7 | 35.0 | 0.51 |
| Beta-blocker | 20.6 | 35.7 | **0.032** | 16.8 | 25.2 | **0.041** |
| Calcium-blocker | 23.6 | 42.9 | **0.009** | 23.8 | 28.2 | 0.34 |
| Diuretic | 28.8 | 40.5 | 0.12 | 28.7 | 31.1 | 0.65 |
| Other | 4.8 | <11.9^†^ | 0.45 | 4.5 | 8.7 | 0.087 |
| Systolic blood pressure (mmHg) | 146±22 | 154±17 | **0.021** | 146±21 | 150±27 | 0.081 |
| Diastolic blood pressure (mmHg) | 81±12 | 82±10 | 0.49 | 81±14 | 80±13 | 0.088 |
| Pulse rate (beats/min) | 70±12 | 74±15 | 0.080 | 70±12 | 72±15 | 0.068 |
| Atrial fibrillation (%) | 3.5 | 12.2 | **0.017** | 3.6 | <4.9^†^ | >0.99 |
| Left ventricular hypertrophy (%) | 2.0 | 0 | >0.99 | 1.6 | 4.9 | **0.042** |
| Lipid-lowering medications (%) | 68.5 | 83.3 | **0.042** | 67.5 | 67.0 | 0.91 |
| Total serum cholesterol (mmol/L) | 4.4±1.1 | 4.4±1.1 | 0.67 | 4.4±1.1 | 4.5±1.3 | 0.37 |
| Serum HDL-cholesterol (mmol/L) | 1.22±0.33 | 1.24±0.35 | 0.79 | 1.23±0.34 | 1.22±0.36 | 0.80 |
| Serum triglycerides (mmol/L)^†^ | 1.5 (0.9-2.6) | 1.8 (1.1-2.8) | 0.090 | 1.5 (0.9-2.6) | 1.6 (0.9-2.8) | 0.37 |
| High sensitivity C-reactive protein (mg/L) | 2.4 (0.8-7.4) | 2.4 (0.8-7.2) | >0.99 | 2.4 (0.8-7.4) | 3.2 (1.1-9.6) | **0.010** |
| Serum vitamin B-12 (pmol/L) | 335 (212-529) | 297 (190-464) | 0.10 | 333 (211-525) | 348 (207-587) | 0.33 |
| Serum bicarbonate (mmol/L) | 24.5±2.3 | 23.6±2.4 | **0.027** | 24.5±2.3 | 24.4±2.5 | 0.57 |
| Plasma NT-proBNP (pmol/L) | 71 (17-297) | 143 (42-488) | **0.002** | 62 (16-237) | 161 (30-871) | **<0.001** |
| Serum albumin (g/L) | 4.4±0.3 | 4.4±0.3 | 0.36 | 4.4±0.3 | 4.3±0.4 | 0.074 |
| Serum gamma glutamyltransferase (U/L) | 30 (10-90) | 31 (14-70) | 0.81 | 30 (10-86) | 30 (7-135) | 0.93 |
| Aspirin use (%) | 36.8 | 56.1 | **0.014** | 34.0 | 41.7 | 0.13 |
| Proton pump inhibitor use (%) | 20.2 | 31.0 | 0.12 | 20.3 | 25.2 | 0.25 |
| Serum uric acid (mmol/L) | 0.34±0.09 | 0.39±0.10 | **<0.001** | 0.34±0.09 | 0.35±0.10 | 0.55 |
| eGFR (CKD-EPI) (ml/min/1.73m^2^) | 81.3±20.7 | 64.8±25.3 | **<0.001** | 82.6±19.8 | 72.6±26.6 | **<0.001** |
| eGFR (CKD-EPI) (%): |  |  | **<0.001** |  |  | **<0.001** |
| ≥90 ml/min/1.73m^2^ | 40.5 | 21.4 |  | 42.5 | 33.0 |  |
| 60-89 ml/min/1.73m^2^ | 45.1 | 38.1 |  | 45.3 | 38.8 |  |
| <60 ml/min/1.73m^2^ | 14.3 | 40.5 |  | 12.2 | 28.2 |  |
| Urinary albumin:creatinine ratio (mg/mmol) | 3.1 (0.8-11.5) | 7.1 (1.3-37.1) | **<0.001** | 2.9 (0.8-10.1) | 6.0 (1.0-37.8) | **<0.001** |
| Peripheral sensory neuropathy (%) | 57.1 | 64.3 | 0.43 | 55.9 | 66.0 | **0.048** |
| Any diabetic retinopathy (%) | 37.3 | 41.5 | 0.62 | 34.9 | 58.4 | **<0.001** |
| Peripheral arterial disease (%) | 20.5 | 35.7 | **0.032** | 19.4 | 35.9 | **<0.001** |
| Cerebrovascular disease (%) | 5.0 | 16.7 | **0.006** | 6.5 | 14.6 | **0.008** |
| Coronary heart disease (%) | 27.4 | 38.1 | 0.16 | 20.3 | 41.7 | **<0.001** |
| Heart failure (%) | 5.4 | <11.9^†^ | 0.28 | 3.1 | 13.6 | **<0.001** |
| Anemia (%) | 10.01 | 21.4 | **0.034** | 8.2 | 18.4 | **0.002** |
| Charlson Comorbidity Index (%): |  |  | 0.065 |  |  | **<0.001** |
| 0 | 78.2 | 64.3 |  | 81.2 | 64.1 |  |
| 1-2 | 15.9 | 23.8 |  | 13.6 | 22.3 |  |
| ≥3 | 5.9 | 11.9 |  | 5.3 | 13.6 |  |
| Daily alcohol consumption (standard drinks of 10 U) | 0.1 [0-1.2] | 0.1 [0-1.5] | 0.74 | 0.1 [0-1.2] | 0.1 [0-0.8] | 0.29 |
| Smoking status (%): |  |  | 0.62 |  |  | **<0.001** |
| Current | 11.5 | <11.9^†^ |  | 10.2 | 22.3 |  |
| Anti-TPO positivity (%) | 5.9 | 7.1 | 0.74 | 6.5 | 3.9 | 0.40 |
| Serum TSH (mIU/L) (%): |  |  | 0.49 |  |  | 0.14 |
| <0.34 | 0.3 | 0 |  | 0.2 | 0 |  |
| 0.34-2.9 | 70.7 | 64.3 |  | 72.4 | 65.0 |  |
| 3.0-5.1 | 23.0 | 26.2 |  | 22.0 | 24.3 |  |
| >5.1 | 6.0 | 9.5 |  | 5.4 | 10.7 |  |
| Serum free T4 (pmol/L) (%): |  |  | 0.55 |  |  | >0.99 |
| <12 | 4.2 | 7.1 |  | 4.2 | 3.9 |  |
| 12-22 | 95.2 | 92.9 |  | 95.2 | 96.1 |  |
| >22 | 0.6 | 0 |  | 0.6 | 0 |  |

Data are presented as percentages, mean±SD (standard deviation), geometric mean (SD range), or median [IQR – interquartile range]; NT-proBNP, N-terminal pro b-type natriuretic peptide; ACE-I, angiotensin-converting enzyme inhibitor; ARB, angiotensin receptor blocker; eGFR, estimated glomerular filtration rate by CKD Epidemiology Collaboration equation; TPO, thyroid peroxidase antibody; TSH, thyrotropin; T4, thyroxine. ^†^Actual percentages suppressed to preserve confidentiality
